# The Role of Ventricular Assist Devices in Patients with Ischemic vs. Non-Ischemic Cardiomyopathy

**DOI:** 10.3390/jpm15060241

**Published:** 2025-06-10

**Authors:** Eglė Rumbinaitė, Dainius Karčiauskas, Grytė Ramantauskaitė, Dovydas Verikas, Gabrielė Žūkaitė, Liucija Rancaitė, Barbora Jociutė, Gintarė Šakalytė, Remigijus Žaliūnas

**Affiliations:** 1 Clinical Department of Cardiology, Lithuanian University of Health Sciences, LT-50161 Kaunas, Lithuania; 2 Clinical Department of Cardiac Surgery, Lithuanian University of Health Sciences, LT-50161 Kaunas, Lithuania; 3 Institute of Cardiology, Lithuanian University of Health Sciences, LT-50162 Kaunas, Lithuania; 4 Laboratory for Automation of Cardiovascular Investigation, Institute of Cardiology, Lithuanian University of Health Sciences, LT-50162 Kaunas, Lithuania; 5 Medical Academy, Lithuanian University of Health Sciences, LT-44307 Kaunas, Lithuaniabarbora.jociute@stud.lsmu.lt (B.J.)

**Keywords:** left ventricular assist device, HeartMate 3, ischemic cardiomyopathy, dilated cardiomyopathy, right heart failure, pulmonary hypertension, early mortality, advanced heart failure

## Abstract

**Background**: The HeartMate 3 (HM3) left ventricular assist device (LVAD) has demonstrated improved clinical outcomes in patients with advanced heart failure (HF). However, the influence of underlying HF etiology—ischemic cardiomyopathy (ICM) versus dilated cardiomyopathy (DCM)—on post-implantation outcomes remains insufficiently characterized. **Objectives:** This paper aims to evaluate early postoperative outcomes following HM3 LVAD implantation in patients with ICM versus DCM and to identify the preoperative hemodynamic and clinical predictors of early mortality and hemodynamic instability. **Methods**: We conducted a retrospective single-center cohort study of 30 patients who underwent HM3 LVAD implantation between 2017 and 2024. Patients were stratified by HF etiology (ICM, *n* = 17; DCM, *n* = 13), and preoperative clinical, echocardiographic, and right heart catheterization data were analyzed. The primary endpoint was 30-day postoperative survival. Secondary endpoints included postoperative hemodynamic stability and the need for vasopressor support. **Results**: Non-survivors (*n* = 13) demonstrated elevated central venous pressure (>16.5 mmHg), mean right ventricular pressure (>31.5 mmHg), and pulmonary vascular resistance (>7.5 Wood units), in addition to higher preoperative creatinine levels and longer cardiopulmonary bypass times. Vasopressor requirement postoperatively was associated with elevated pre-implant systolic pulmonary artery pressure. **Conclusions**: Preoperative right-sided pressures and renal dysfunction are strong predictors of early mortality following HM3 LVAD implantation. Patients with ICM exhibit greater early left ventricular recovery compared to those with DCM. These findings underscore the importance of comprehensive and personalized preoperative risk stratification—particularly in patients with DCM and pulmonary hypertension—to optimize postoperative outcomes and guide patient selection for durable LVAD support.

## 1. Introduction

End-stage heart failure (HF) is a major cause of morbidity and mortality all over the world [1] that is characterized by the heart’s inability to maintain adequate blood circulation, resulting in compromised tissue perfusion. As the prevalence of end-stage HF continues to rise, particularly with the aging population [2], it poses significant challenges to healthcare systems. For patients with end-stage HF, conventional treatments and lifestyle changes are often inadequate to maintain long-term survival or improve quality of life. In such instances, advanced therapeutic interventions, including heart transplantation (HTx) and left ventricular assist devices (LVADs), become relevant management strategies. However, due to the limited donor availability, HTx is not always a viable option for patients with end-stage HF. Consequently, there is an increased reliance on LVADs, particularly the HeartMate 3 (HM3) [3], either as a bridge to HTx or, in some cases, as destination therapy for long-term support [4] in patients who are ineligible for HTx. The most relevant function of the HM3 LVAD is to provide mechanical support to the left ventricle (LV) and to alleviate the workload on the heart [5]; this effect can lead to improvements in LV dimensions (remodeling) and functions and contribute to the restoration of hemodynamic stability. Enhanced systemic perfusion resulting from HM3 LVAD support positively impacts end-organ function and improves clinical outcomes associated with end-stage HF [6,7].

By providing robust efficacy in both short- and long-term hemodynamic stabilization, the HM3 LVAD has proven to be a key therapeutic intervention for managing symptoms, prolonging patient survival [8,9,10] and enhancing health-related quality of life in individuals with end-stage HF.

Despite substantial advancements in LVAD therapy, several clinically significant aspects remain inadequately investigated. Among these is the nuanced impact of LVAD support on echocardiographic parameters, especially in the context of distinct HF phenotypes, such as ischemic cardiomyopathy (ICM) and dilated cardiomyopathy (DCM). These two phenotypes have different pathophysiologies that may affect responses to mechanical circulatory support, highlighting the need for stratified analyses to guide patient selection and predict individual risks.

Accordingly, the present study seeks to investigate the role of preoperative clinical and echocardiographic variables in predicting postoperative hemodynamic instability and all-cause mortality among recipients of the HM3 LVAD, with particular attention to differences between ICM and DCM cohorts.

## 2. Materials and Methods

### 2.1. Patients

This retrospective, single-center cohort study included 30 consecutive patients who underwent HM3 LVAD implantation between 2017 and 2024. All relevant preoperative assessments were conducted following institutional protocols, and the indication for HM3 LVAD implantation was determined based on predefined criteria according to the most recent clinical guidelines issued by the European Society of Cardiology (ESC). The decision for HM3 LVAD implantation was made by the local “Heart team” consilium accordingly. No exclusion criteria were applied, allowing for an inclusive representation of the patient population undergoing advanced HF management at the center. Informed consent was obtained from all participants, and ethical approval for the study was granted by the institutional review board. Clinical data were extracted from electronic medical records, operative reports, and echocardiographic evaluations. All data were anonymized and stored per data protection regulations, ensuring complete confidentiality and the absence of any identifiable patient information.

### 2.2. Preoperative Assessment and Data Collection

Demographic, clinical, and laboratory data were retrospectively collected for all patients before HM3 LVAD implantation. The preoperative variables extracted from medical records included age, sex, body mass index (BMI), etiology, and the duration of HF and documented comorbidities. Standard hematologic and biochemical profiles were obtained, with all values interpreted following institutional reference ranges. All patients underwent intraoperative transesophageal echocardiographic (TEE) assessment before and after HM3 LVAD implantation to guide device positioning and evaluate immediate structural and functional cardiac changes. Transthoracic echocardiography (TTE) was performed for all patients prior to discharge, seven to ten days following HM3 LVAD implantation during LVAD support, with the same imaging modality. The echocardiography protocol included the evaluation of LV end-diastolic and end-systolic dimensions; LV ejection fraction (LVEF); LV mass; left atrial (LA) diameter and volume; right ventricular (RV) dimensions and systolic function—including tricuspid annular plane systolic excursion (TAPSE) and RV systolic velocity (RV S′)—pulmonary artery (PA) diameter; and PA acceleration time. It is important to mention that echocardiography results might vary because of image reproducibility and interobserver variability.

Hemodynamic assessment was conducted preoperatively via pulmonary artery catheterization. The parameters recorded included central venous pressure (CVP); systolic, diastolic, and mean RV pressures; systolic, diastolic, and mean PA pressures; pulmonary capillary wedge pressure (PCWP); mean arterial pressure (MAP); cardiac output (CO); cardiac index (CI); and pulmonary vascular resistance (PVR). The cardiopulmonary bypass (CPB) time was extracted from intraoperative surgical records. The use of vasopressors was recorded during the first seven postoperative days. Thirty-day survival following HM3 LVAD implantation was documented as the primary early outcome measure.

### 2.3. Statistical Analysis

Descriptive statistics were presented as means ± standard deviations (SDs) for continuous variables and absolute counts with corresponding percentages for categorical variables. Comparative statistical analyses were performed using the Student’s *t*-test for parametric variables and the Mann–Whitney U test for non-parametric distributions. The study population was divided into groups for comparative analysis based on HF etiology (ICM vs. DCM) and 30-day postoperative survival statuses (follow-up calls and registries) to evaluate the potential preoperative predictors of mortality. Additionally, patients were stratified based on the requirement for vasopressor support within the first seven days following HM3 LVAD implantation (vasopressor-dependent vs. vasopressor-independent) to investigate the potential association between preoperative pulmonary hypertension and early postoperative hemodynamic instability. Receiver operating characteristic (ROC) curve analysis was employed to calculate the area under the curve (AUC) and optimal threshold values, sensitivity, and specificity for the selected variables predictive of early postoperative mortality. A two-sided *p*-value of <0.05 was considered statistically significant. All analyses were conducted using IBM SPSS Statistics, version 29 (IBM Corp., Armonk, NY, USA).

## 3. Results

### 3.1. Patient Characteristics

A total of 30 patients who underwent HM3 LVAD implantation between 2017 and 2024 were included in the study cohort. The population comprised 83.3% males (*n* = 25) and 16.7% females (*n* = 5), with a mean age of 58 years (range: 27–67 years) and a mean body weight of 81.9 kg (range: 60–113 kg) at the time of implantation.

The predominant etiology of end-stage HF was ICM, accounting for 53.3% of cases (*n* = 16), followed by DCM in 33.3% (*n* = 10) and HF secondary to valvular heart disease in 13.3% (*n* = 4). The average duration of HF before HM3 implantation was 5.5 years (range: 0–10 years). During the 30-day follow-up period, 13 patients (43.3%) died following HM3 implantation. The remaining 17 patients (56.7%) continue to be monitored, with no mortality observed during the ongoing follow-up.

### 3.2. Comparative Analysis of Ischemic Cardiomyopathy and Dilated Cardiomyopathy Groups

To evaluate the potential impact of HF etiology on preoperative and surgical characteristics, the study cohort was divided into two groups based on the underlying cause of end-stage HF: ICM and DCM. Comparative analysis was performed to assess whether the origin of HF was associated with significant differences in clinical presentation or perioperative variables. Table 1 compares selected preoperative laboratory values, echocardiographic measurements, and intraoperative parameters between the ICM and DCM groups.

Preoperative laboratory parameters did not differ significantly between the ICM and DCM groups, with both cohorts exhibiting markedly elevated N-terminal pro b-type natriuretic peptide (NT-proBNP) levels, consistent with advanced HF. TTE performed prior to HM3 LVAD implantation revealed severely reduced left and right ventricular systolic function, accompanied by biventricular and biatrial chamber dilatation in both groups. Post-implantation echocardiographic assessment demonstrated a statistically significant improvement in LVEF among patients with ICM compared to those with DCM. Specifically, the median LVEF increased to 36% [IQR: 16–59] in the ICM group versus 9% [IQR: 6–14] in the DCM group (*p* = 0.036), suggesting differential patterns of ventricular recovery based on underlying HF etiology.

### 3.3. Association Between Preoperative Predictors and Postoperative Mortality

Another objective of this study was to identify pre-implantation parameters that may be associated with increased mortality risk following HM3 LVAD implantation (presented in Table 2).

### 3.4. Hemodynamic Predictors of Early Postoperative Mortality

**Figure 1:**
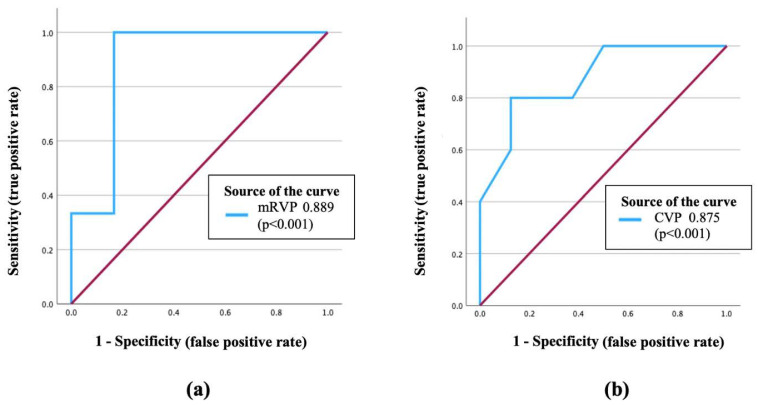
Receiver operating characteristic (ROC) curves of (**a**) median right ventricular pressure and (**b**) central venous pressure. AUC for median right ventricular pressure is 0.889 (*p* < 0.001) and 0.875 for central venous pressure (*p* < 0.001). CVP—Central venous pressure; mRVP—median right ventricular pressure.

Among the preoperative hemodynamic variables evaluated, CVP and median right ventricular pressure (mRVP) emerged as the most robust predictors of early postoperative mortality following HM3 LVAD implantation. Both parameters demonstrated high discriminative power, with AUC values of 0.875 and 0.889, respectively, and were statistically significant (*p* < 0.001 for both). These findings were supported by strong sensitivity and specificity values, indicating their potential clinical utility in identifying patients at elevated risk. Pulmonary vascular resistance (PVR) was also significantly associated with increased mortality risk, yielding an AUC of 0.811 (*p* = 0.041). Notably, a PVR threshold exceeding 7.5 Wood units was identified as a critical cut-off associated with adverse postoperative outcomes. In contrast, other parameters—including diastolic RV pressure, systolic pulmonary artery pressure (sPAP), CO, and CI—did not demonstrate significant predictive value. ROC curve analysis was performed to evaluate the prognostic accuracy of CVP and mean RV pressure (mRVP) as predictors of postoperative mortality. As shown in Figure 1, among the hemodynamic variables analyzed, both CVP and mRVP demonstrated significant predictive value. These results suggest that elevated CVP, RV pressure, and PVR are key pre-implantation hemodynamic markers associated with early mortality in patients undergoing HM3 LVAD support.

### 3.5. Preoperative Echocardiographic and Surgical Parameters Associated with Mortality

To further explore potential preoperative predictors of postoperative instability and mortality (30-day), the cohort was stratified into survivors (*n* = 17) and non-survivors (*n* = 13). Table 3 presents a comparative analysis of echocardiographic, hemodynamic, and intraoperative variables between these groups.

### 3.6. Clinical and Perioperative Characteristics Associated with Mortality

In the non-survivor cohort (*n* = 13), the follow-up period extended to 30 days post-implantation, with four cases of in-hospital mortality documented. The comparative analysis of postoperative outcomes revealed that non-survivors exhibited a significantly higher BMI, with median values increasing from 23.9 [IQR: 22.6–24.7] kg/m^2^ in survivors to 29.4 [IQR: 25.4–31.5] kg/m^2^ in non-survivors (*p* = 0.007). Serum creatinine levels were also markedly elevated among non-survivors (124.0 (110–163) μmol/L) compared to survivors (83.0 (65–115) μmol/L, *p* = 0.007), indicating the presence of preexisting renal dysfunction. In terms of hemodynamic and intraoperative parameters, CVP was significantly higher in the non-survivor group (27 (14–30) mmHg) relative to survivors (9 (7–17) mmHg, *p* = 0.03), suggesting a greater degree of venous congestion. Additionally, prolonged CPB time was associated with mortality, with median durations increasing from 102 (82–111) minutes in survivors to 123 (104–128]) min in non-survivors (*p* = 0.011). No statistically significant differences were observed between the two groups in other preoperative or perioperative parameters. Taken together, these findings suggest that comorbidities, such as renal impairment, elevated BMI, elevated CVP (indicative of systemic congestion), and prolonged CPB time, may serve as important contributors to early mortality following HM3 LVAD implantation.

### 3.7. Pulmonary Artery Pressure as a Marker of Postoperative Hemodynamic Instability Following HeartMate 3 LVAD Implantation

To evaluate preoperative echocardiographic characteristics potentially associated with prolonged postoperative hemodynamic instability following HM3 LVAD implantation, patients were stratified based on vasopressor requirements during the first seven postoperative days. Two groups were defined: those requiring vasopressor support (*n* = 12, V^+^ group) and those who remained vasopressor-independent (*n* = 18, V^−^ group). The median duration of HF did not significantly differ between the groups (five years in the V^−^ group vs. six years in the V^+^ group). Similarly, CPB times were comparable, with median durations of 102 min in the V^−^ group and 111 min in the V^+^ group (*p* = 0.246), indicating no significant procedural time discrepancy. A detailed comparison of preoperative biomarkers and standard echocardiographic parameters across both groups is provided in Table 4, aiming to identify early imaging and biochemical indicators of postoperative circulatory instability.

### 3.8. Pulmonary Hypertension and Postoperative Hemodynamic Instability

Preoperative laboratory parameters did not significantly differ between the vasopressor-dependent (V^+^) and vasopressor-independent (V^−^) groups. However, a significant difference was observed in preoperative sPAP, which was notably higher in patients who required vasopressors following HM3 implantation (median: 61 mmHg [IQR: 55–69.5]) compared to those who did not (median: 46 mmHg [IQR: 44–NA]; *p* = 0.036). This finding suggests that moderate pulmonary hypertension, as assessed noninvasively before surgery, may serve as a predictor of sustained postoperative hemodynamic instability.

No other echocardiographic parameters demonstrated statistically significant differences between the two groups. These results indicate that elevated preoperative sPAP may identify patients at increased risk for prolonged circulatory support needs, highlighting the prognostic relevance of pulmonary hypertension in the perioperative assessment of LVAD candidates.

## 4. Discussion

### 4.1. Differences Between Ischemic and Dilated Cardiomyopathy in End-Stage Heart Failure

LVADs are an important treatment strategy for patients with end-stage HF, particularly in cases of a bridge to transplantation strategy. However, outcomes following LVAD implantation may vary depending on the underlying etiology of HF, though this association remains incompletely understood. In the present study, we compared outcomes between patients with end-stage HF of ICM and DCM origins—two of the most prevalent HF phenotypes [11]. It is important to mention that this is a single-center study, and the comparison between groups might underestimate subtle differences, limited to a quite small cohort stratified into groups. Also, the statistical power might be limited due to a relatively small sample size, and the results should be assessed under the consideration of these limitations.

Post-implantation echocardiographic analysis revealed a more substantial improvement in LVEF among ICM patients compared to their DCM counterparts (36% [IQR: 16–59] vs. 9% [IQR: 6–14]; *p* = 0.036). Importantly, the baseline LVEF did not differ significantly between the two cohorts, suggesting a different myocardial response to mechanical unloading.

According to the existing literature, patients with ICM may demonstrate a more favorable response to LVAD therapy, potentially due to the reduced involvement of the RV compared to individuals with non-ischemic cardiomyopathy (non-ICM) [12]. Furthermore, evidence supports the LVAD-induced reverse remodeling of the LV in patients with ICM, an effect that appears less pronounced or absent in those with DCM [13].

However, the findings on long-term outcomes remain inconclusive. A recent systematic meta-analysis comparing ICM and non-ICM patients undergoing LVAD implantation reported no statistically significant differences in early postoperative outcomes, including one-year survival, suggesting that HF etiology may not be an independent determinant of survival in this context [14].

In contrast, data from the European Registry for Patients with Mechanical Circulatory Support (EUROMACS) indicated higher early mortality rates among patients with ICM compared to those with idiopathic cardiomyopathy. Notably, this observed difference was substantially influenced by baseline covariates such as age and comorbidity burden, underscoring the need for risk-adjusted analyses in interpreting outcome disparities between HF subtypes [15]. Similarly, findings from a single-center study in Germany, which assessed both short-term and long-term outcomes in patients with ICM versus DCM post-LVAD implantation, revealed no significant differences between the two groups—a result consistent with broader trends reported in the literature [16].

Addressing long-term follow-up, patients who had non-ICM were more likely to recover in the International Registry for Patients with Mechanical Circulatory Support (INTERMACS) analysis in the bridge to recovery group, taken together with other favorable outcome factors—younger age and a shorter duration of HF [17]. Another single-center study also revealed no significant differences between 30-day, 6-month, and 12-month mortality rates in ICM vs. non-ICM groups [18]. The analysis of the TriNetX database showed better long-term survival in DCM patients compared to ICM patients [19]. However, in our study, there is no data on long-term survival rates and long-term prognostic predictors; this limits the relevance and provides an opportunity for future studies.

### 4.2. Predictors of Early Mortality Following HeartMate 3 Left Ventricular Assist Device Implantation

In the context of advanced HF, risk stratification plays a critical role in guiding therapeutic decisions, particularly when considering LVAD implantation. The present study identified several preoperative and perioperative parameters associated with early postoperative mortality following HM3 implantation, with particular emphasis on markers indicative of right heart failure (RHF). These established parameters could improve personalized decision-making for end-stage HF patients and predict their outcomes, making this decision more individualized and achieving a better prognosis for each patient.

Elevated CVP (>16.5 mmHg), increased mRVP (>31.5 mmHg), and PVR (>7.5 Wood units) emerged as strong predictors of early mortality. These hemodynamic markers collectively reflect compromised RV function and elevated right-sided filling pressures—markers of RHF. Our findings align closely with the EUROMACS data, demonstrating that non-survivors exhibited higher right-sided pressures, increased PVR, and elevated right atrial-to-pulmonary capillary wedge pressure ratios. When combined with clinical evidence of fluid retention, these metrics serve as reliable indicators of RHF and correlate with adverse postoperative outcomes [20].

The pathophysiological relevance of PVR in this context is well-established, as elevated PVR imposes a greater afterload on the RV, thereby exacerbating right-sided dysfunction and increasing the risk of early postoperative decompensation [21]. Notably, RHF is a frequent complication following LVAD implantation, with reported incidence rates ranging from 5% to 46% [22]. Its presence is associated with markedly reduced survival and worsened quality of life [23]. As such, the identification of RHF prior to implantation may be vital for prognostication and tailoring perioperative individual management strategies aimed at RV preservation.

In addition to hemodynamic factors, several clinical and intraoperative variables were also associated with increased early mortality. These included higher BMI, elevated serum creatinine concentrations, and prolonged CPB time. A retrospective study of the EUROMACS registry showed that obesity (BMI 30 kg/m^2^ and over) at the time of LVAD implantation is associated with significantly higher mortality and increased risk of infection [24]. Our findings suggest that even sub-threshold elevations in BMI may confer increased risk, consistent with prior reports [20].

Renal dysfunction also emerged as a relevant prognostic factor. Elevated creatinine levels, reflective of impaired renal reserve, have been associated with worse LVAD outcomes in multiple studies [20,23,25]. This relationship is likely mediated by the bidirectional pathophysiological interplay between renal dysfunction and RHF, wherein elevated venous pressures compromise renal perfusion and reduced renal function exacerbates volume overload and RV strain [26,27].

Finally, a longer CPB duration was significantly associated with early mortality in our cohort. This observation is consistent with previous evidence linking prolonged operative times to heightened risks of postoperative RHF and systemic complications [21]. The CPB time may reflect both the technical complexity of the implantation procedure and the physiological burden imposed on an already compromised circulatory system.

Taken together, these findings underscore the multifactorial nature of early mortality risk following HM3 implantation, with particular emphasis on right-sided heart function, renal reserve, and procedural burden. The early identification and mitigation of these factors may help identify these patients and improve postoperative outcomes in this high-risk population.

## 5. Conclusions

Preoperative right-sided pressures and renal dysfunction are strong predictors of early mortality following HM3 LVAD implantation. Patients with ICM exhibit greater early LV recovery than those with DCM. These findings underscore the importance of comprehensive preoperative risk stratification, particularly in patients with DCM and pulmonary hypertension, in optimizing postoperative outcomes and guiding patient selection for durable LVAD support.

## Figures and Tables

**Table 1:** Comparison of preoperative and postoperative parameters between ICM and DCM groups.

Variables	ICM Patients N = 16	DCM Patients N = 10	*p*-Value
**Laboratory findings**			
Hemoglobin, g/L	122.0 (99.0; 140.0)	129.0 (112.0; 151.0)	0.50
White blood count, 10^9^/L	7.8 (6.0; 10.5)	7.0 (4.8; 13.2)	0.70
Thrombocytes, 10^9^/L	205.0 (158.0; 269.0)	198.0 (134.0; 270.0)	0.50
CRP, mg/L	22.0 (4.0; 37.0)	15.0 (5.0; 45.0)	0.80
Potassium, mmol/L	4.0 (3.9; 4.3)	4.0 (3.4; 4.4)	0.70
Sodium, mmol/L	134.0 (131.0; 139.0)	137.0 (128.0; 138.0)	1.00
Creatinine, μmol/L	116.0 (86.0; 128.0)	94.0 (65.0; 136.0)	0.50
AST, IU/L	31.0 (20.0; 78.0)	35.0 (22.0; 162.0)	0.70
ALT, IU/L	34.0 (19.0; 96.0)	41.0 (14.0; 158.0)	0.80
GGT, IU/L	65.0 (34.0; 180.0)	125.0 (42.0; 174.0)	1.00
NT-proBNP, ng/L	4122.0 (2228.0; 9441.0)	3154.0 (2059.0; 4277.0)	0.40
**Echocardiographic parameters**			
LV end-diastolic diameter, mm	72.0 (63.0; 76.0)	71.0 (64.0; 74.0)	0.90
Interventricular septum, mm	9.0 (9.0; 10.0)	8.0 (8.0; 10.0)	0.20
LV posterior wall, mm	8.0 (7.0; 9.0)	10.0 (9.0; 10.0)	0.06
LV mass, g	264.0 (244.0; 352.0)	301.0 (255.0; 324.0)	1.00
LV ejection fraction, %	15.0 (12.0; 17.0)	10.0 (10.0; 16.0)	0.10
LA diameter, mm	53.0 (51.0; 57.0)	58.0 (48.0; 67.0)	0.30
RV end-diastolic diameter, mm	50.0 (45.0; 56.0)	52.0 (50.0; 55.0)	0.50
PA diameter, mm	29.0 (26.0; 31.0)	31.0 (26.0; 37.0)	0.60
PA acceleration time, ms	77.0 (60.0; 80.0)	72.0 (64.0; 78.0)	0.60
TAPSE, mm	14.0 (11.0; 17.0)	13.0 (8.0; 15.0)	0.40
RV S′, cm/s	6.7 (4.5; 8.0)	6.6 (5.5; 10.5)	0.50
**Surgical parameters**			
CPB time, min	102.0 (83.0; 126.0)	111.0 (92.0; 124.0)	0.80

ALT—Alanine transaminase; AST—aspartate transaminase; CBP—cardiopulmonary bypass; CRP—C-reactive protein; DCM—dilated cardiomyopathy; GGT—γ-glutamyltransferase; ICM—ischemic cardiomyopathy; LA—left atrium; LV—left ventricular; NT-proBNP—N-terminal pro b-type natriuretic peptide; PA—pulmonary artery; RV—right ventricular; S′—systolic excursion velocity; TAPSE—tricuspid annular plane systolic excursion.

**Table 2:** Preoperative hemodynamic parameters associated with early mortality following HeartMate 3 left ventricle assisting device implantation.

	AUC	*p*-Value	Cut-Off	Sensitivity	Specificity
Central Venous Pressure, mmHg	0.875	**<0.001**	16.5	80.0	87.5
Systolic Right Ventricular Pressure, mmHg	0.764	**0.040**	52.0	75.0	78.0
Diastolic Right Ventricular Pressure, mmHg	0.500	1.000			
Median Right Ventricular Pressure, mmHg	0.889	**<0.001**	31.5	100.0	83.0
Systolic Pulmonary Artery Pressure, mmHg	0.500	1.000			
Diastolic Pulmonary Artery Pressure, mmHg	0.750	0.200			
Median Pulmonary Artery Pressure, mmHg	0.639	0.400			
PCWP, mmHg	0.694	0.200			
Mean Arterial Blood Pressure, mmHg	0.667	0.500			
Cardiac Output, L/min	0.542	0.800			
Cardiac Index, L/min/m^2^	0.625	0.400			
Pulmonary Vascular Resistance, Wood Units	0.811	**0.040**	7.5	80.0	89.0

AUC—Area under the curve; PCWP—pulmonary capillary wedge pressure. The bolded *p*-values are statistically significant.

**Table 3:** Parameters between the survivor vs. non-survivor study groups.

	Survivors	Non-Survivors	*p*-Value
	(*n* = 17)	(*n* = 13)	
**BMI, kg/m^2^**	23.9 (22.6; 24.7)	29.4 (25.4; 31.5)	**0.007**
**Laboratory Findings**			
Hemoglobin, g/L	127.0 (116.0; 152.0)	129.0 (100.0; 145.0)	0.600
White blood count, 10^9^/L	7.8 (6.0; 11.3)	6.9 (5.2; 9.5)	0.300
Thrombocytes, 10^9^/L	240.0 (188.0; 264.0)	158.0 (141.0; 249.0)	0.200
CRP, mg/L	16.0 (5.0; 41.0)	11.0 (3.0; 29.0)	0.900
Potassium, mmol/L	4.3 (4.0; 4.5)	4.1 (3.9; 4.2)	0.300
Sodium, mmol/L	133.0 (130.0; 137.0)	138.0 (133.0; 139.0)	0.100
Creatinine, μmol/L	83.0 (65.0; 115.0)	124.0 (110.0; 163.0)	**0.007**
AST, IU/L	43.0 (22.0; 60.0)	31.0 (22.0; 81.0)	0.600
ALT, IU/L	39.0 (18.0; 87.0)	38.0 (22.0; 45.0)	0.900
GGT, IU/L	125.0 (64.0; 208.0)	56.0 (30.0; 139.0)	0.100
NT-proBNP, ng/L	2948.0 (1764.0; 5464.0)	5074.0 (1968.0; 8419.0)	0.400
**Instrumentally Assessed Parameters**			
LV end-diastolic diameter, mm	70.0 (62.0; 78.0)	72.0 (65.0; 76.0)	0.500
Interventricular septum, mm	9.0 (8.0; 10.0)	9.5 (9.0; 10.3)	0.200
LV posterior wall, mm	9.0 (8.2; 9.8)	8.5 (7.0; 9.6)	0.400
LV mass, g	282.0 (245.0; 314.0)	303.0 (242.0; 352.0)	0.500
LV ejection fraction, %	15.0 (12.0; 20.0)	12.0 (10.0; 15.0)	0.100
LV end-diastolic volume, mL	256.0 (134.0; 306.0)	263.0 (247.0; 294.0)	1.000
LA diameter, mm	55.0 (53.0; 66.0)	56.0 (51.0; 58.0)	0.500
LA volume, mL	167.0 (119.0; 246.0)	150.0 (140.0; 251.0)	1.000
RV end-diastolic diameter, mm	48.0 (44.0; 51.0)	52.0 (51.0; 58.0)	0.060
TAPSE, mm	13.0 (9.5; 16.0)	14.0 (10.0; 16.0)	0.800
RV S‘, cm/s	6.0 (4.0; 9.0)	6.3 (4.0; 8.0)	0.800
TA diameter, mm	43.0 (32.0; 48.0)	44.0 (34.0; 50.0)	1.000
PA diameter, mm	28.0 (26.0; 33.0)	29.0 (27.0; 31.0)	1.000
PA acceleration time, ms	74.0 (64.0; 79.0)	77.0 (67.0; 95.0)	0.400
Systolic PAP, mmHg	55.0 (46.0; 67.0)	54.0 (45.0; 69.0)	1.000
Mean PAP, mmHg	41.0 (40.0; 49.0)	43.0 (40.0; 48.0)	0.800
Central venous pressure, mmHg	9.0 (7.0; 17.0)	27.0 (14.0; 30.0)	**0.030**
PVR, Wood units	4.0 (3.9; 4.8)	11.0 (6.0; 18.0)	0.060
**Surgical Parameters**			
CPB time, min	102.0 (82.0; 111.0)	123.0 (104.0; 128.0)	**0.010**

ALT—Alanine transaminase; AST—aspartate transaminase; BMI—body mass index; CPB—cardiopulmonary bypass time; CRP—C-reactive protein; GGT—γ-glutamyltransferase; LA—left atrium; LV—left ventricular; NT-proBNP—N-terminal pro b-type natriuretic peptide; PA—pulmonary artery; PAP—pulmonary artery pressure.; PVR—pulmonary vascular resistance; RV—right ventricular; TA—tricuspid annulus; TAPSE—tricuspid annular plane systolic excursion. The bolded *p*-values are statistically significant.

**Table 4:** Preoperative study characteristics between the two study groups.

	V^−^ (*n* = 18)	V^+^ (*n* = 12)	*p*-Value
**Laboratory Findings**			
Potassium, mmol/L	4.2 (4.0; 4.4)	3.9 (3.5; 4.6)	0.90
Hemoglobin, g/L	129.0 (117.0; 150.0)	127.0 (100.0; 134.0)	0.50
White blood count, 10^9^/L	7.4 (6.0; 11.1)	7.9 (5.5; 10.0)	0.90
Thrombocytes, 10^9^/L	249.0 (148.0; 264.0)	188.0 (115.0; 234.0)	0.20
CRP, mg/L	10.0 (2.0; 22.0)	31.0 (5.0; 57.0)	0.08
Sodium, mmol/L	137.0 (132.0; 138.0)	134.0 (122.0; 138.0)	0.40
Creatinine, μmol/L	105.0 (69.0; 127.0)	108.0 (87.0; 140.0)	0.40
AST, IU/L	31.0 (18.0; 49.0)	43.0 (31.0; 97.0)	0.20
ALT, IU/L	34.0 (16.0; 48.0)	45.0 (32.0; 87.0)	0.20
GGT, IU/L	115.0 (51.0; 164.0)	65.0 (42.0; 204.0)	0.90
NT-proBNP (ng/L)	3183.0 (2336.0; 8598.0)	3265.0 (511.0; 6301.0)	0.40
**Echocardiographic Parameters**			
LV end-diastolic diameter, mm	70.5 (66.1; 75.0)	67.0 (60.3; 76.0)	0.40
Interventricular septum, mm	9.0 (8.5; 10.0)	9.0 (8.0; 10.0)	0.90
LV posterior wall, mm	9.0 (8.0; 9.4)	9.0 (8.0; 10.0)	0.50
Myocardial mass index, g/m^2^	282.0 (261.0; 324.0)	258.0 (228.0; 348.0)	0.60
LV ejection fraction, %	14.0 (10.0; 15.0)	15.0 (12.0; 19.0)	0.30
LV end-diastolic volume, mL	263.0 (228.0; 305.0)	211.0 (134.0; 369.0)	1.00
LV end-systolic volume, mL	226.0 (203.0; 256.0)	146.0 (102.0; 216.0)	0.10
LA diameter, mm	54.0 (51.0; 67.0)	55.0 (53.0; 59.0)	1.00
LA volume, mL	216.0 (128.0; 281.0)	140.0 (127.0; 167.0)	0.17
RV end-diastolic diameter, mm	50.0 (44.0; 55.0)	52.0 (48.0; 53.0)	0.40
TAPSE, mm	13.0 (12.0; 16.0)	11.5 (7.8; 15.5)	0.30
RV S ′, cm/s	6.6 (5.3; 8.5)	6.0 (3.0; 8.0)	0.40
TA diameter, mm	43.0 (37.0; 49.0)	33.0 (13.0; 46.0)	0.40
PA diameter, mm	29.0 (26.0; 33.0)	28.0 (26.0; 31.0)	0.70
PA acceleration time, ms	72.0 (61.0; 79.5)	76.5 (70.0; 83.0)	0.40
systolic PAP, mmHg	61.0 (55.0; 70.0)	46.0 (44.0; 49.0)	**0.03**
mean PAP, mmHg	41.0 (40.0; 47.5)	43.5 (40.0; 48.0)	1.00

ALT—Alanine transaminase; AST—aspartate transaminase; CRP—C-reactive protein; GGT—γ-glutamyltransferase; LA—left atrium; LV—left ventricular; NT-proBNP—N-terminal pro b-type natriuretic peptide; RV—right ventricular; PA—pulmonary artery; PAP—pulmonary artery pressure; S′—systolic excursion velocity; TA—tricuspid annulus; TAPSE—tricuspid annular plane systolic excursion. The bolded *p*-values are statistically significant.

## Data Availability

The data presented in this study are available upon request from the corresponding author for ethical reasons.
